# Sodium Bicarbonate Decreases Alcohol Consumption in Mice

**DOI:** 10.3390/ijms25095006

**Published:** 2024-05-03

**Authors:** Jason Lin, Ana P. Rivadeneira, Yani Ye, Clara Ryu, Shangrila Parvin, Kyeongran Jang, Sandra M. Garraway, Inyeong Choi

**Affiliations:** Department of Cell Biology, Emory University School of Medicine, Atlanta, GA 30322, USA; jasonlin7710@gmail.com (J.L.); yani.ye@emory.edu (Y.Y.); clara.ryu@emory.edu (C.R.); sparvin@emory.edu (S.P.); kjang8@emory.edu (K.J.); smgarraway@emory.edu (S.M.G.)

**Keywords:** alcohol consumption, pH, sodium bicarbonate, alcohol drinking, alcoholism

## Abstract

We previously reported that mice with low neuronal pH drink more alcohol, demonstrating the importance of pH for alcohol reward and motivation. In this study, we tested whether systemic pH affects alcohol consumption and if so, whether it occurs by changing the alcohol reward. C57BL/6J mice were given NaHCO_3_ to raise their blood pH, and the animals’ alcohol consumption was measured in the drinking-in-the-dark and two-bottle free choice paradigms. Alcohol consumption was also assessed after suppressing the bitterness of NaHCO_3_ with sucrose. Alcohol reward was evaluated using a conditioned place preference. In addition, taste sensitivity was assessed by determining quinine and sucrose preference. The results revealed that a pH increase by NaHCO_3_ caused mice to decrease their alcohol consumption. The decrease in high alcohol contents (20%) was significant and observed at different ages, as well as in both males and females. Alcohol consumption was also decreased after suppressing NaHCO_3_ bitterness. Oral gavage of NaHCO_3_ did not alter quinine and sucrose preference. In the conditioned place preference, NaHCO_3_-treated mice spent less time in the alcohol-injected chamber. Conclusively, the results show that raising systemic pH with NaHCO_3_ decreases alcohol consumption, as it decreases the alcohol reward value.

## 1. Introduction

Excessive alcohol drinking has devastating health consequences [1,2]. Between 2011 and 2015, excessive drinking was responsible for more than 95,000 deaths and an average of 29 years lost per death in the US each year [3], and the death rate increased by 39% in 2021 [4]. Excessive drinking is associated with alcohol-use disorder (AUD), which causes 3 million deaths worldwide, according to the World Health Organization [5]. Preventing excessive use of alcohol is, thus, essential for reducing the risk of AUD. The Centers for Disease Control and Prevention recommends cutting back on drinking, and many people benefit simply by controlling their habits by drinking slowly, choosing alcohol-free days, or setting a limit on how much they will drink [6]. However, maintaining new drinking habits is not an easy task, and some people regain old habits later. Medical treatment approved by the US Food and Drug Administration is prescribed only to people who have already stopped drinking and are trying to maintain abstinence [7]. In this regard, developing a non-medication strategy that can easily reduce excessive alcohol drinking will be valuable for people at risk of developing AUD and for others who want to drink moderately.

pH is an important biological factor in the body, as H^+^ binds to proteins and alters the structure and function of bound proteins [8,9,10,11]. In the nervous system, pH affects the activities of many proteins, such as receptors, ion channels, transporters, and signaling proteins, thus resulting in physiological and pathological consequences [12,13,14]. A small amount of H^+^ or ${\mathrm{HCO}}_{3}^{-}$ moving across cell membranes can cause a large pH change at the synapses, where the synaptic cleft is typically closed off; as a result, synaptic activity and the complex firing pattern can be altered. In this sense, it is conceivable that pH may alter alcohol modulatory properties in the brain, such that alcohol-drinking frequency and amount are changed. For example, pH can affect dopamine transmission [15,16], which is responsible for motivation and reward [17]. pH also alters the activities of γ-aminobutyric acid (GABA) receptors and N-methyl-D-aspartate (NMDA) receptors, both of which play key roles in synaptic plasticity relevant to the development of addictive behaviors [18,19,20].

We previously reported that alcohol consumption is high in mice with targeted inactivation of a pH-regulating protein found in many cells, including neurons [21]. These mice have low pH in neurons and display increased responses to alcohol due to the development of increased motivation to drink alcohol. These findings led us to test whether alcohol consumption could be affected by systemic pH and, if so, whether it would occur by changing the alcohol reward value. In this study, we raised blood pH in C57BL/6 mice by orally administering NaHCO_3_ and analyzed the animals’ alcohol consumption, effective dose of NaHCO_3_, effective alcohol content, and taste sensitivity. The effect of NaHCO_3_ on alcohol reward was also evaluated. The results show that mildly increasing blood pH with NaHCO_3_ decreases alcohol consumption, as it decreases the alcohol reward value.

## 2. Results

### 2.1. Acute Ingestion of NaHCO_3_ Decreases Alcohol Consumption in Mice

**Figure 1:**
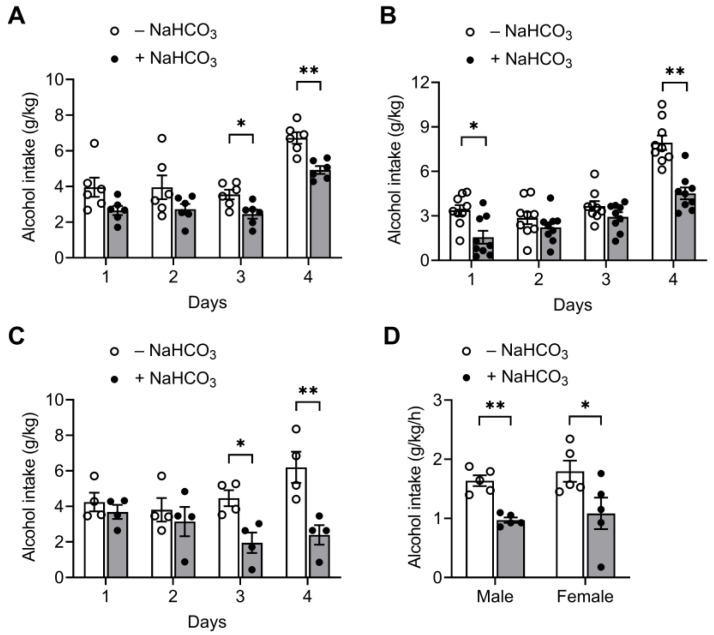
Alcohol consumption before and after oral gavage of NaHCO_3_. Mice at 2 months (8 weeks) (**A**), 4 months (**B**), and 14 months (**C**) were given oral gavage of NaHCO_3_ (0.25 g/kg body weight) or water and, 1 h later, allowed free access to 20% alcohol. The alcohol-drinking sessions were 2 h on days 1–3 and 4 h on day 4. Alcohol consumption was measured as g/kg body weight. (**D**) Male vs. female. Alcohol consumption per hour on days 1–4 was compared. * *p* < 0.05, ** *p* < 0.01.

Table 1 shows the results of blood chemistry in C57BL/6J mice, measured at 0, 1, and 2 h after oral gavage of NaHCO_3_ (0.25 g/kg body weight). Blood pH increased from 7.35 to 7.44 at 1 h and 7.47 at 2 h (*p* < 0.05; one-way ANOVA with repeated measures). These changes are comparable to blood pH increases at 1–2 h after ingestion of similar amounts of NaHCO_3_ in humans [22,23,24]. As expected, ${\mathrm{HCO}}_{3}^{-}$ and base excess (i.e., an excess in the amount of base) increased, while PCO_2_ remained unchanged. In parallel experiments, a different cohort of mice was given a NaHCO_3_ gavage and, one hour later, allowed free access to 20% alcohol in the DID paradigm. The control mice were given water instead of NaHCO_3_. As shown in Figure 1, NaHCO_3_-treated mice (i.e., animals with raised blood pH) decreased their alcohol consumption, compared with water-treated controls (*p* < 0.05; two-way ANOVA). The decrease was particularly prominent on day 4, when alcohol drinking was allowed for 4 h. The decrease was observed in mice at different ages, as well as males and females, indicating that the effect of NaHCO_3_ on alcohol consumption occurs regardless of age and sex.

### 2.2. Chronic Ingestion of NaHCO_3_ Decreases Alcohol Consumption and Preference in Mice

**Figure 2:**
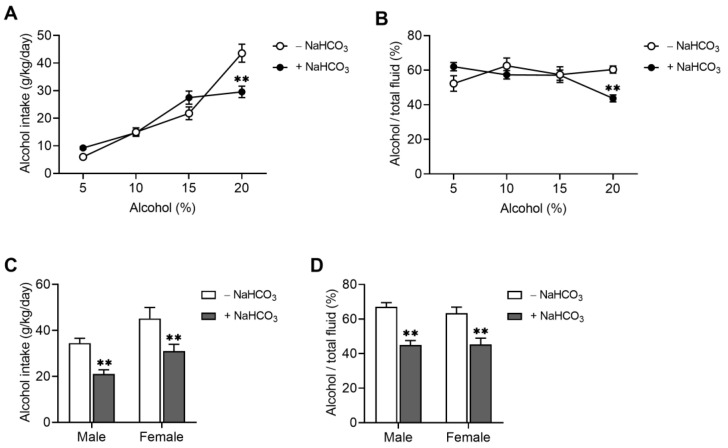
Effects of NaHCO_3_ on alcohol consumption in mice. (**A**) Effects of NaHCO_3_ on the consumption of 5–20% alcohol contents in the two-bottle free choice paradigm. Mice (3–4 months) were allowed free access to 5–20% alcohol for 4 days in two-bottle free choice (*n* = 10). Mice were then allowed free access to 0.3 M NaHCO_3_ for 6 days, and alcohol consumption was measured again in the continued presence of NaHCO_3_. Measurements were performed daily at each concentration. (**B**) Alcohol preference (i.e., alcohol intake/total fluid intake). (**C**,**D**) Comparison of alcohol consumption (**C**) and preference (**D**) between males and females. Consumption of 20% alcohol was compared (*n* = 11 males and 7 females). ** *p* < 0.01.

The DID procedure we used in the above experiment is useful to assess the effects of pharmacological compounds (NaHCO_3_ in our study) on binge-like alcohol consumption, especially when the action of a drug is short term [25]. On the other hand, DID is limited in testing some other properties of a drug, such as its effects on alcohol preference or taste sensitivity. For this reason, we measured alcohol consumption in mice using the two-bottle free choice paradigm in which animals were allowed free access to two bottles: one containing water and the other containing alcohol. Blood pH was raised by allowing mice free access to 0.3 M NaHCO_3_ for 6 days and maintained during the experiment. As shown in Table 2, chronic ingestion of NaHCO_3_ increased blood pH by 0.19 pH units (*p* < 0.05; Student *t*-test). As expected, ${\mathrm{HCO}}_{3}^{-}$ and base excess increased while most of the other parameters, including PCO_2_, anion gap, BUN, hematocrit, and hemoglobin, remained unchanged. NaHCO_3_ decreased blood K^+^ level (*p* < 0.05), which is consistent with hypokalemia in metabolic alkalosis [26,27]. Figure 2A shows the comparison of 5–20% alcohol consumption before and after NaHCO_3_ ingestion. The consumption of 5–15% alcohol was similar between the two groups, but the consumption of 20% alcohol was significantly decreased after NaHCO_3_ ingestion (*p* < 0.05 for the interaction between alcohol content and NaHCO_3_; two-way ANOVA). Consistent with this result, there was a decrease in alcohol preference (i.e., ratio of alcohol intake–total fluid intake) at 20% (Figure 2B). These changes were observed in both males and females (Figure 2C,D), again consistent with negligible sex difference, as shown in the above DID.

### 2.3. NaHCO_3_ at the Concentrations of 0.1–0.4 M Decreases Alcohol Consumption without Causing Diarrhea

**Figure 3:**
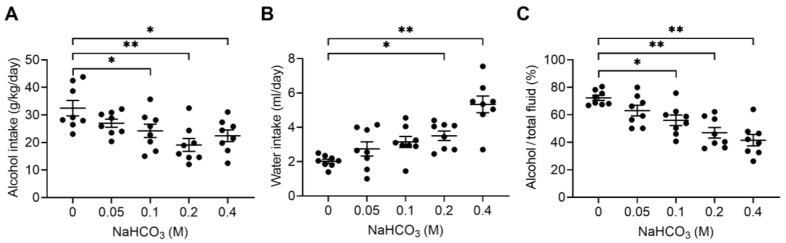
Dose-dependent effects of NaHCO_3_. (**A**) Alcohol consumption at different concentrations of NaHCO_3_. Mice were given access to the two-bottle free choice with water and 20% alcohol in the presence of 0.05–0.4 M NaHCO_3_. Both bottles contained NaHCO_3_ to minimize a potential tastant effect. (**B**) Consumption of NaHCO_3_-containing water. (**C**) Alcohol preference. * *p* < 0.05, ** *p* < 0.01.

**Figure 4:**
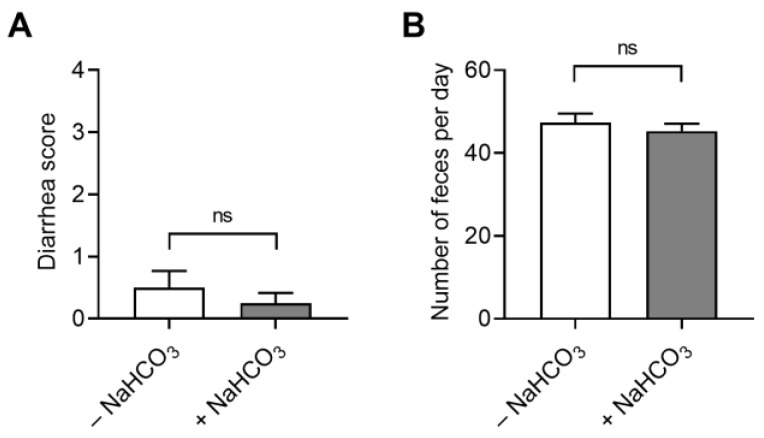
Effects of NaHCO_3_ on diarrhea. (**A**) Diarrhea scores. Feces were collected from mice before and after 0.4 M NaHCO_3_ administration for 2 days, and diarrhea scores were counted based on watery conditions (*n* = 8/group). Scores were scaled using the following stool conditions: 0: normal, 1: soft stool, 2: slight wet and soft stool, 3: wet and unformed stool with moderate perianal staining on the coat, and 4: watery stool with severe perianal staining on the coat. (**B**) Number of feces produced daily before and after NaHCO_3_ ingestion (*n* = 10). ns: not significant.

To determine the dose-dependent effects of NaHCO_3_ on alcohol consumption, mice were subjected to the two-bottle free choice with water and 20% alcohol in the presence of 0.05–0.4 M NaHCO_3_. As shown in Figure 3A, alcohol consumption began to decrease at a concentration of 0.1 M and higher (*p* < 0.05; one-way ANOVA with repeated measures). Calculated from the fluid volume ingested, the minimum effective dose of NaHCO_3_ corresponded to 0.05 g/kg body weight. Mice increased the consumption of water containing NaHCO_3_, as shown in Figure 3B. This inverse relationship resulted in a progressive decrease in alcohol preference as NaHCO_3_ concentrations increased (Figure 3C). The highest concentration in our study was 0.4 M, which can cause gastrointestinal distress, such as diarrhea in humans [28]. We examined the condition of feces produced by mice to determine whether the animals had diarrhea. We found no significant difference in diarrhea scores between NaHCO_3_-treated mice and water-treated mice (Figure 4A). The number of feces produced by the two groups was also similar (Figure 4B). Thus, drinking up to 0.4 M NaHCO_3_ decreased alcohol consumption without causing diarrhea in our study.

### 2.4. Decreased Alcohol Consumption Is Not Due to the Unpleasant Taste of NaHCO_3_

**Figure 5:**
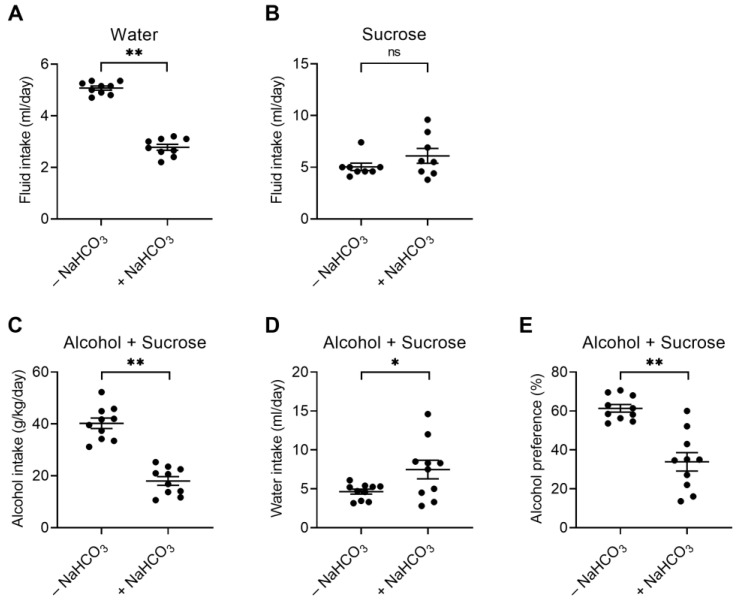
Alcohol consumption after the suppression of NaHCO_3_ bitterness by sucrose. (**A**) Fluid consumption in the absence and presence of NaHCO_3_. The measurements were done before and after 0.3 M NaHCO_3_ ingestion in a two-bottle free choice (*n* = 8). (**B**) Suppression of the bitterness of NaHCO_3_ by sucrose. Fluid consumption was measured as shown in (**A**) in the presence of 1% sucrose. (**C**–**E**) Effects of NaHCO_3_ on alcohol consumption (**C**), water consumption (**D**), and alcohol preference (**E**) in the presence of 1% sucrose (*n* = 10). * *p* < 0.05, ** *p* < 0.01, ns: not significant.

NaHCO_3_ has a bitter and metallic taste and is unpleasant to drink. In the above dose–response experiments, alcohol consumption was more profoundly decreased at higher NaHCO_3_ concentrations. While this result supports the importance of high pH for decreasing alcohol consumption, an alternative explanation is that mice might have consumed less fluid due to the unpleasant taste of NaHCO_3_. We conducted two sets of experiments to address this possibility. First, mice were allowed free access to NaHCO_3_ in the presence of sucrose, which suppresses the bitter taste [29]. Figure 5A shows the baseline consumption of pure water vs. NaHCO_3_-containing water in a two-bottle free choice. Mice did not prefer NaHCO_3_-containing water to pure water. In the presence of 1% sucrose, however, mice consumed NaHCO_3_-containing water as much as pure water (*p* > 0.05; Student *t*-test) (Figure 5B), indicating the suppression of NaHCO_3_ bitterness by sucrose. Next, alcohol consumption was measured in the presence of sucrose and compared before and after 6 days of NaHCO_3_ ingestion. As shown in Figure 5C–E, mice with NaHCO_3_ ingestion decreased alcohol consumption and preference, while increasing NaHCO_3_-containing water. These changes were comparable to those in the absence of sucrose (Figure 3). Thus, the ability of NaHCO_3_ to decrease alcohol consumption occurred regardless of sucrose, indicating that the unpleasant taste of NaHCO_3_ has a negligible impact on decreasing alcohol consumption.

### 2.5. NaHCO_3_ Can Decrease Alcohol Consumption without Altering Taste Sensitivity

**Figure 6:**
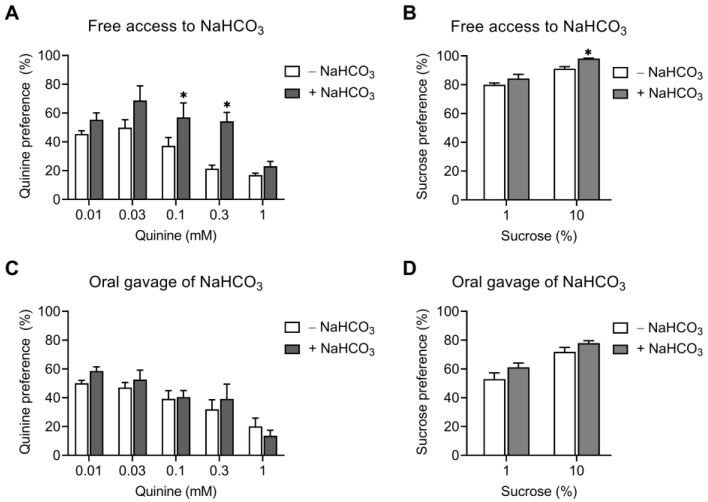
Effects of NaHCO_3_ on quinine and sucrose sensitivity. (**A**,**B**) Quinine and sucrose sensitivity in mice with free access to NaHCO_3_. Mice were offered 0.01–1 mM quinine hydrochloride and water in a two-bottle choice, and quinine consumption was measured before and after 6 days of ingestion of NaHCO_3_. Both bottles contained NaHCO_3_ when measured after NaHCO_3_ ingestion. Each concentration was offered for 24 h. Quinine preference was determined as a ratio of quinine intake to total fluid intake (*n* = 5 for each concentration except 10 for 0.01 mM). For sucrose sensitivity, the procedure was similar to that in (**A**), except that 1% and 10% (*w*/*v*) sucrose solutions were used (*n* = 8). (**C**,**D**) Quinine and sucrose sensitivity in mice with oral gavage of NaHCO_3_. Mice were given NaHCO_3_ or water by oral gavage and, 1 h later, offered 0.01–1 mM quinine hydrochloride or 1% and 10% sucrose solutions. Mice were allowed to drink for 4 h in the DID procedure (*n* = 5/group). * *p* < 0.05.

To determine the effect of NaHCO_3_ on taste sensitivity, mice were allowed free access to 0.01–0.3 mM quinine and water in the two-bottle choice, and quinine consumption was measured before and after 6 days of NaHCO_3_ ingestion. Both bottles contained NaHCO_3_ when measured after NaHCO_3_ ingestion. NaHCO_3_ caused quinine preference to increase (*p* < 0.05; two-way ANOVA), as shown in Figure 6A. The increase was significant at 0.1 and 0.3 mM. In separate experiments, mice were given access to 1% and 10% sucrose and water, and sucrose consumption was measured before and after NaHCO_3_ ingestion. No difference was found in 1% sucrose preference, whereas there was an increase in 10% sucrose preference (Figure 6B). Thus, free access to NaHCO_3_ appears to influence taste sensitivity.

In this experiment, mice were given free access to NaHCO_3_, such that taste buds on the tongue were influenced by NaHCO_3_. This led to a question of whether the effect of NaHCO_3_ on alcohol consumption would be mediated by raising the blood pH or by directly changing taste sensitivity on the tongue. To address this question, we gave mice NaHCO_3_ by oral gavage to deliver NaHCO_3_ directly to the stomach and assessed quinine and sucrose preference. As shown in Figure 6C, both NaHCO_3_-treated mice and water-treated controls had a similar quinine preference (*p* > 0.05; two-way ANOVA). Both groups also had a similar sucrose preference (*p* > 0.05; two-way ANOVA) (Figure 6D). Thus, oral gavage of NaHCO_3_ had negligible impacts on taste sensitivity, indicating that the effect of NaHCO_3_ on alcohol consumption can occur without altering taste sensitivity.

### 2.6. NaHCO_3_ Decreases Alcohol-Reinforcing Effects

**Figure 7:**
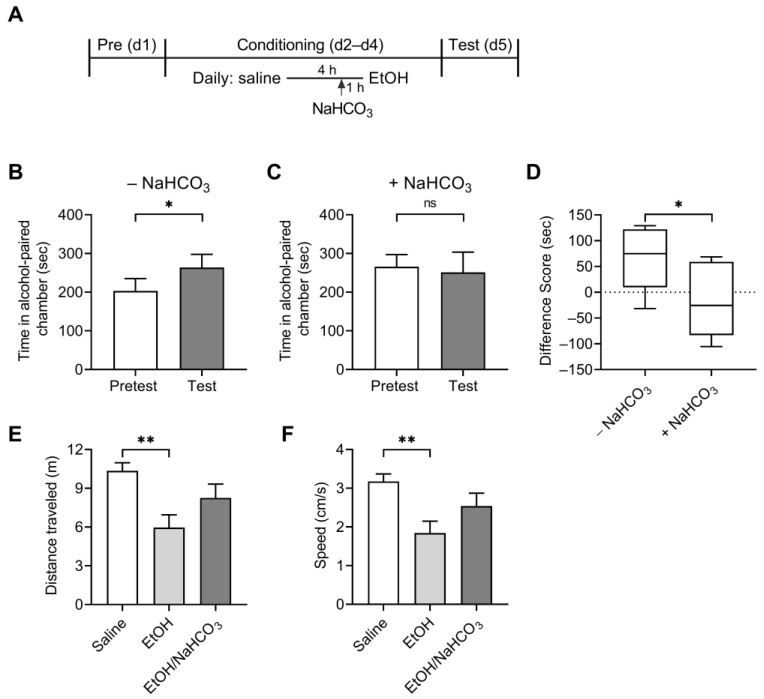
Effects of NaHCO_3_ on alcohol reward. (**A**) Timeline of experimental treatment and behavioral measurements in alcohol-induced CPP. Following the 15 min pretest, 5 min conditioning sessions were conducted daily for 3 days, with saline injection in the morning and 2 g/kg alcohol injection 4 h later. One group of mice received a gavage of 0.25 g/kg NaHCO_3_ one hour prior to alcohol injection, while another group of mice received a gavage of water (*n* = 7–8/group). In the test session, mice were allowed to explore the chambers for 15 min. (**B**,**C**) Time spent in the alcohol-paired chamber during the pretest and test sessions in control mice (**B**) and NaHCO_3_-treated mice (**C**). (**D**) CPP scores. Scores were calculated by subtracting the time spent in the alcohol-paired chamber in the pretest from that in the test. (**E**) Distance traveled during the conditioning session. Travel distance was measured for 5 min after injection of saline or alcohol and averaged. (**F**) Speed (cm/s). * *p* < 0.05, ** *p* < 0.01, ns: not significant.

Decreased voluntary alcohol consumption can be driven by either a decreased reward value of alcohol, thus leading to reduced motivation for alcohol consumption, or an increased reward value of alcohol, thus requiring less alcohol to achieve the same level of hedonic response [30,31]. We examined the reinforcing effects of alcohol using CPP in which mice received oral gavage of NaHCO_3_ or water 1 h before alcohol conditioning (Figure 7A). Water-treated control mice increased the time spent in the alcohol-injected chamber after conditioning (Figure 7B), consistent with a place preference conditioned by alcohol. In contrast, NaHCO_3_-treated mice showed negligible increase after conditioning (*p* > 0.05; Student *t*-test), as shown in Figure 7C. NaHCO_3_ gavage alone without alcohol injection had no effect (Supplementary Data S1). The difference between the two groups of mice was evident when the CPP scores were calculated (Figure 7D). The scores were significantly decreased in NaHCO_3_-treated mice, indicating a decreased alcohol reward value. Alcohol decreased the distance traveled and the speed when measured during conditioning (*p* < 0.05 for each; one-way ANOVA) (Figure 7E,F). This effect was blunted in NaHCO_3_-treated mice. Conclusively, the CPP results demonstrate that NaHCO_3_ decreases the reinforcing effects of alcohol.

## 3. Discussion

The major findings in this study are highlighted as follows. (i) A systemic pH increase by NaHCO_3_ ingestion decreases alcohol consumption in mice. (ii) This effect is evident with higher alcohol contents and occurs in mice at different ages, as well as in both males and females. (iii) The unpleasant taste of NaHCO_3_ is not involved in decreasing alcohol consumption. (iv) Decreased alcohol consumption is driven by decreased alcohol-reinforcing effects, thus reducing the motivation to drink alcohol. (v) NaHCO_3_ can decrease alcohol consumption without altering taste sensitivity. The significance of our study is that we identified pH as a biological factor that can influence alcohol-drinking properties. In alcohol research, pH change has been viewed as the consequence of alcohol metabolism, given that chronic and excessive alcohol drinking causes lactic acidosis and ketoacidosis [32,33]. No study has been done to examine whether pH conversely affects alcohol drinking. In this sense, our study, for the first time, provides evidence for the role of pH in modulating alcohol reward and drinking properties and presents a different paradigm from the current understanding. Considering that a small pH difference has been considered minor in function and is often ignored, our observation of marked changes in alcohol consumption by a 0.1 pH unit increase is, thus, interesting.

We raised the blood pH in mice using NaHCO_3_. In humans, NaHCO_3_ ingestion improves pH, [${\mathrm{HCO}}_{3}^{-}$], base excess, and PCO_2_ compared to the placebo condition [34]. ${\mathrm{HCO}}_{3}^{-}$ loading is ergogenic in sporting events that heavily generate energy through anaerobic glycolysis and challenge acid–base homeostasis [35,36]. Ingestion of 0.2–0.3 g/kg NaHCO_3_ increases blood pH by 0.05–0.1 pH unit for 1–3 h [22,23,24]. Consistent with these changes, we observed a comparable increase in blood pH after oral gavage of 0.25 g/kg NaHCO_3_ (Table 1). This amount transiently increased blood [${\mathrm{HCO}}_{3}^{-}$] by 5–6 mM. In humans, 0.3 g/kg NaHCO_3_ causes bloating and diarrhea in some studies, while the same amount has no effect in other studies. At much higher doses, NaHCO_3_ can cause abnormal electrical activity, volume expansion, and hypernatremia [37]. Healthy adults can tolerate up to 1700 mEq (142.8 g) NaHCO_3_ daily [38,39]. This corresponds to 2.1 g/kg NaHCO_3_ in mice. In our study, we used 0.25 g/kg NaHCO_3_ for oral gavage, markedly lower than the tolerance level. NaHCO_3_ is removed from the kidneys or converted to CO_2_ and H_2_O, and the addition of new ${\mathrm{HCO}}_{3}^{-}$ is temporary and unlikely to cause a severe metabolic complication.

We found that mildly raised blood pH by NaHCO_3_ caused mice to consume less alcohol. This effect was observed in acute and chronic ingestions, as well as by voluntary (DID and two-bottle free choice) and involuntary (gavage) drinking paradigms. Thus, the pH change, not the procedure to administer NaHCO_3_, is responsible for decreasing alcohol consumption. The decrease was profound with the alcohol content of 20% while being negligible with the lower contents (Figure 3). Thus, pH probably affects the efficacy of alcohol to produce the maximum response, but not its potency to increase consumption. Drug efficacy is the intrinsic ability of a drug to activate its target molecule, whereas drug potency is a binding affinity between a drug and its target molecule [40]. Thus, we think that pH affects alcohol’s ability to activate, or inactivate, its target proteins (receptors) but has no effect on the rate of the binding between alcohol and its target proteins. Because efficacy is an intrinsic characteristic of alcohol, it is likely that pH alters the pharmacological properties of the target proteins, resulting in a decreased maximum response to alcohol. This interpretation is consistent with the fact that pH can alter protein structure and function and modulate the relevant synaptic activities and complex firing patterns in the brain.

An interesting finding from our study is the increase in the consumption of NaHCO_3_-containing water in the presence of alcohol (Figure 3B). Mice did not prefer NaHCO_3_-containing water to pure water when the two solutions were given in the two-bottle free choice. This is understandable, as NaHCO_3_ has a bitter and salty taste. However, when sucrose was provided, mice consumed NaHCO_3_-containing water as much as pure water and, more importantly, consumed less alcohol. Thus, the alcohol consumption decrease caused by NaHCO_3_ is unrelated to its taste. This interpretation leads to a possibility that alcohol may abolish the bitterness of NaHCO_3_. Alcohol has a sweet component in addition to a bitter component in animals [41] and humans [42]. Furthermore, the relationship between sucrose and alcohol intake has been documented [43,44]. A cross-sectional study by Liu et al. [43] revealed that many heavy drinkers have taste dysfunction, determined by failing to correctly identify the bitter taste of quinine in the whole-mouth test. Silva et al. [44] observed low sensitivity for sweet taste in alcoholics. Excessive alcohol drinking can change the sensitivity of taste receptors and compromise the functions of taste. Together, we think that, similar to sucrose, alcohol may suppress NaHCO_3_ bitterness, and this suppressive property of alcohol facilitates the consumption of NaHCO_3_-containing water.

Regarding the effect of NaHCO_3_ on taste sensitivity, quinine and sucrose preferences were similar between NaHCO_3_-treated mice and pair-treated mice, when NaHCO_3_ was given by oral gavage. However, these preferences were different between the two groups when mice were allowed free access to NaHCO_3_ (Figure 6). Oral gavage allows NaHCO_3_ to be directly delivered to the stomach with minimum contact with taste buds on the tongue. The lack of change in the preferences, thus, indicates that the decreasing effect of NaHCO_3_ on alcohol consumption can occur without changing taste sensitivity. Given that NaHCO_3_ reduces alcohol consumption regardless of the administration procedures, we think that the two properties are not closely related to each other. Additional studies are required to further assess the effect of NaHCO_3_ on taste sensitivity.

The results from the current study are consistent with our previous finding that alcohol consumption is enhanced in NBCn1 knockout mice [21]. These mice have decreased resting pH in neurons and cerebrospinal fluid; thus, they develop acidic brain pH. Increased alcohol consumption in these mice is due neither to a difference in taste sensitivity, as there is no significant effect of genotype on quinine and sucrose preference, nor to alcohol clearance, as blood alcohol contents are similar between genotypes. Instead, it is due to the increased reward value of alcohol because the knockout mice develop an increased propensity for the alcohol-induced conditioned place preference. The knockout mice also have an increased tolerance to alcohol-induced sedative effects. We did not test whether NaHCO_3_ affects a tolerance to alcohol-induced sedation.

What would be the underlying mechanism of pH-dependent changes in alcohol consumption? The neural circuit that plays a key role in alcohol reward is the mesolimbic pathway [31,45], in which alcohol stimulates dopamine (DA) release from the ventral tegmental area (VTA) nerve terminals in the nucleus accumbens (NAc) in the ventral striatum. The stimulation occurs, in part, by decreasing GABAergic inhibition in the VTA, which results in a disinhibition of DA neuronal activity [17]. The stimulation also occurs by affecting glutamate release from various glutamatergic projections that feed into the VTA and NAc and mediate the synaptic plasticity relevant to addictive behaviors [18,19]. GABA_A_ receptors have variable pH sensitivity due to the different pharmacological and biophysical properties of their subunits [46]. NMDA receptors are also sensitive to physiological pH (IC50 = pH 7.3), and their pH dependence has been a model for pH-mediated regulation of neuronal activity [47]. It is unclear whether a similar model is involved in the mesolimbic pathway, but we note that a 0.3 pH unit change alters the current in the NAc [48]. Furthermore, pH may influence dopamine release and uptake [15], dopamine autoxidation [16], and its binding to dopamine receptors [49]. pH may cause significant effects on the dopaminergic transmission associated with alcohol motivation and reward, subsequently changing alcohol consumption. In this regard, we note that postmortem tissue of people with AUD has low brain pH, compared with controls [50,51].

In summary, our study demonstrates the importance of systemic pH for alcohol consumption and alcohol reward. The results lead to the possibility that NaHCO_3_ as a mediator has the potential to decrease excessive alcohol consumption in humans and improve alcohol-related behaviors. NaHCO_3_ is a dietary supplement and has a lower risk of adverse effects. Furthermore, NaHCO_3_ can enhance the anti-inflammatory response [52,53], thus suggesting that NaHCO_3_ not only reduces alcohol consumption but may also protect the body from alcohol-induced peripheral insults. Taken together, we propose that temporarily raising blood pH by NaHCO_3_ can be a useful approach to reducing excessive alcohol consumption and ultimately reducing the risk of AUD.

## 4. Materials and Methods

### 4.1. Animals

This study was conducted in accordance with the National Institute of Health Guide for the Care and Use of Laboratory Animals, approved by the Institutional Animal Care and Use Committee at Emory University. C57BL/6J male and female mice at the age of 12–24 weeks were purchased from Jackson Labs (Bar Harbor, ME, USA) and housed on a 12 h light–dark cycle. Mice were provided with standard chow and water ad libitum. Blood chemistry was analyzed using an i-STAT 1 blood analyzer (Abbott Laboratories, Abbott Park, IL, USA) with EC8+ and CG8+ cartridges.

### 4.2. Drinking in the Dark (DID)

DID was conducted using the protocol by Thiele et al. [54] with the following modification. One week before the initiation of testing, mice were individually housed in the reverse light-cycle room and given standard mouse chow and water ad libitum. Each mouse was weighed before DID. During the first 3 h of the dark phase, mice were given oral gavage of NaHCO_3_ (0.25 g/kg body weight) 1 h prior to alcohol drinking. The gavage volume was 10 × body weight (µL/g). The controls were water gavage. The mice were allowed to drink 20% alcohol for 2 h on days 1–3 and 4 h on day 4 to achieve an alcohol binge, as described by Thiele et al. [54]. Alcohol consumption was determined per body weight (g/kg body weight).

### 4.3. Two-Bottle Free Choice

Mice were individually housed and allowed continuous access to two bottles: one bottle containing water and another bottle containing alcohol with various concentrations of 5–20% alcohol (*v*/*v*). Each concentration was offered for 4 days. Alcohol consumption (g/kg body weight) was calculated by subtracting the volume of the solutions left in the bottles from the initial volume. Measurement was made daily at each concentration and averaged. The bottles were switched every day to minimize a potential side preference. To increase blood pH, the mice were given free access to a solution containing 0.2–0.3 M NaHCO_3_ for 6 days, which gradually increases blood pH and stably maintains a raised pH during alcohol-consumption experiments. The mice were then subjected to an alcohol-consumption assessment in the two-bottle free choice. NaHCO_3_ was added to both bottles to maintain increased blood pH. For the experiments with the suppression of NaHCO_3_ bitterness, 1% sucrose was added to both bottles. Alcohol preference was calculated from the ratio of alcohol intake to total fluid intake (alcohol + water). Alcohol evaporates over time and the evaporation rate varies with different alcohol contents. Our preliminary test showed 6.2–13.1% evaporation in 24 h for 5–20% alcohol contents, and the changes were similar, regardless of NaHCO_3_ in the solutions. The loss by evaporation was not subtracted in this study, as we compared alcohol consumption between the control group and the test group.

### 4.4. Diarrhea Assessment

Diarrhea was assessed using the diarrhea scoring system by Sakai et al. [55]. Scores were scaled based on the following stool conditions, 0: normal, 1: soft stool, 2: slight wet and soft stool, 3: wet and unformed stool with moderate perianal staining on the coat, and 4: watery stool with severe perianal staining on the coat. The assessment was conducted with mice that had been given 0.4 M NaHCO_3_ (the highest concentration in the two-bottle free choice) for 2 days. The average score and the incidence of each diarrhea score were obtained.

### 4.5. Taste Sensitivity

Taste sensitivity was assessed in two different ways. First, the assessment was made in the two-bottle free choice paradigm as described previously [21]. The mice were individually housed and offered two bottles containing water and 0.01–1 mM of quinine hydrochloride. Each quinine concentration was offered for 1 day; in between each quinine test day, the animals were given two bottles of water for 2 days. The bottles were weighed daily and switched. Then, the mice were given free access to 0.3 M NaHCO_3_ for 6 days, and the quinine experiments were repeated. NaHCO_3_ was added to both bottles to maintain an increased blood pH. For sucrose preference, 1% or 10% (*w*/*v*) of sucrose was used instead of quinine. Quinine or sucrose preference was determined as a ratio of taste solution intake to total fluid intake. Second, taste sensitivity was assessed in the DID paradigm. The mice were given an oral gavage of NaHCO_3_ (0.25 g/kg) or water during the first 3 h of the dark phase and then allowed to drink 0.01–1 mM of quinine hydrochloride for 4 h. Each concentration was offered for 1 day. For sucrose preference, 1% or 10% (*w*/*v*) of sucrose was used instead of quinine. Quinine or sucrose preference was determined as a ratio of taste solution intake to total fluid intake.

### 4.6. Conditioned Place Preference (CPP)

Alcohol-induced CPP was performed as described previously [56] with a slight modification. Mice were acclimated to the testing room for at least several hours a day before testing. The CPP apparatus had two chambers: one with white walls and the other with black walls separated by a small partition [57]. On day 1 (pretest), mice were given full access to the chambers for 15 min. The chamber for placing mice was random and alternated. Following the pretest, 5 min conditioning sessions were conducted daily for 3 days, with saline injections in the morning and 2 g/kg alcohol injections 4 h later. One group of mice received oral gavage of 0.25 g/kg NaHCO_3_ one hour prior to alcohol injection, and another group of mice received water instead of NaHCO_3_. The gavage was performed in the home cages to eliminate potential preference/aversion caused by NaHCO_3_ or the gavage itself. Conditioning was performed using an unbiased design [58], in which the animals were placed in the black chamber after saline injection and in the white chamber after alcohol injection. On day 5 (test), the mice were placed into the chamber where they were initially placed in the pretest and allowed free access to the apparatus for 15 min. The time spent in the alcohol-paired chamber was measured and compared to the time spent in the same chamber in the pretest. The results were compared between NaHCO_3_-treated and water-treated mice. For testing the effect of NaHCO_3_ on alcohol-related locomotor activity, distance traveled and speed in the conditioning sessions were analyzed. All experiments were recorded using an overhead camera and analyzed using the tracking software Toxtrac version 2.98 [59].

### 4.7. Statistical Analysis

Data were reported as mean ± standard error of the mean. A two-tailed, unpaired Student *t*-test was used when analyzing diarrhea scores and CPP scores and a two-tailed, paired test when analyzing blood chemistry before and after NaHCO_3_ administration and suppression of the bitterness with sucrose. One-way ANOVA with repeated measures was used when analyzing blood chemistry at different time points after NaHCO_3_ gavage and the effective dose of NaHCO_3_, and an ordinary one-way ANOVA was used when analyzing time traveled and speed in CPP. A two-way ANOVA with a Sidak post hoc test was used when analyzing alcohol consumption in DID, different alcohol contents in the two-bottle free choice, sex difference, and taste sensitivity. A *p* value of <0.05 was considered significant. The analysis was made using Prism 9 (GraphPad; La Jolla, CA, USA) and Microsoft Office Excel add-in Analysis ToolPak (Redmond, WA, USA). Outliers were determined using Grubbs’ test.

## Figures and Tables

**Table 1:** Blood chemistry before and after oral gavage of NaHCO_3_ (0.25 g/kg body weight).

	Time after NaHCO_3_ Gavage		
Parameters	0 h	1 h	2 h
pH	7.35	7.44 *	7.47 **
HCO_3_, mM	21.6	25.8 *	25.4 **
BE, mM	−3.8	3.4 *	1.4 **
PCO_2_, mmHg	39.8	33.2	34.8

BE: base excess, PCO_2_: partial CO_2_ pressure; * *p* < 0.05, ** *p* < 0.01, *n* = 5.

**Table 2:** Blood chemistry before and after 6-day access to 0.3 M NaHCO_3_.

	WT	
Parameters	−NaHCO_3_	+NaHCO_3_
pH	7.2	7.39 *
HCO_3_, mM	17.7	29.4 *
PCO_2_, mmHg	43.6	48.2
BE, mM	−10.1	4.2 *
Na, mM	151	155
K, mM	6.1	3.5 *
Cl, mM	118	114
BUN, mg/dL	23.9	26.6
HCt, %PCV	48	47
Hb, g/dL	16.3	16

BE: base excess, PCO_2_: partial CO_2_ pressure, BUN: blood urea nitrogen, HCt, hematocrit, PCV: packed cell volume, Hb: hemoglobin, * *p* < 0.05, *n* = 4.

## Data Availability

The data presented in this study are available upon request to I.C.

## Supplementary Materials

The supporting information can be downloaded at: https://www.mdpi.com/article/10.3390/ijms25095006/s1.
