# Supplementary material for: Sodium Bicarbonate Decreases Alcohol Consumption in Mice
Source: Int J Mol Sci. 2024 May 3;25(9):5006. doi: 10.3390/ijms25095006 (PMC11084513; doi:10.3390/ijms25095006)
Supplement: Supplementary file 1 [file ijms-25-05006-s001.zip › ijms-2926850-supplementary.pdf]

Supplementary Data S1

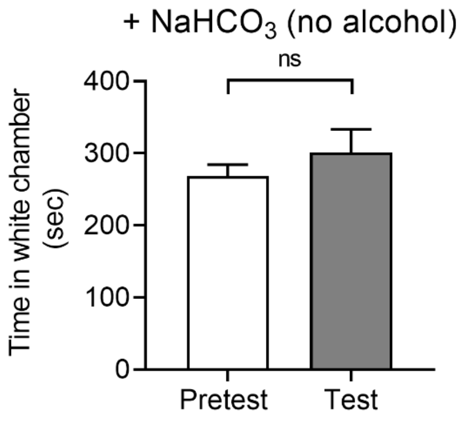

Negligible effect of $NaHCO_3$ alone on conditioned place preference. Method: During the pretest (day 1), mice were given full access to the chambers for 15 min. The chamber for placing mice was random and alternated. On conditioning sessions (days 2–4), mice were given $NaHCO_3$ (0.25 g/kg body weight) or water by oral gavage in the home cages and one hour later allowed full access to the CPP chambers for 5 min. The chamber for placing mice was the chamber where alcohol was assigned to be given. On the test day (day 5), mice were allowed free access to the chambers for 15 min. Result: No significant difference in the time spent in the alcohol-assigned chamber between pretest and test (p > 0.5, n = 5; Student t-test).
